# A de novo derivative Y chromosome (partial Yq deletion and partial duplication of Yp and Yq) in a female with disorders of sex development

**DOI:** 10.1002/ccr3.1613

**Published:** 2018-07-07

**Authors:** Qing‐Song Liu, Xing‐Chun Zhu, Qiang Ma, Cheng He, Jian‐Lan Shao

**Affiliations:** ^1^ Department of Clinical Laboratory Affiliated Hospital of North Sichuan Medical College Nanchong Sichuan China; ^2^ Pathogenic Biology and Immunology Experiment Teaching Center North Sichuan Medical College Nanchong Sichuan China; ^3^ Department of Obstetrics and Gynecology Affiliated Hospital of North Sichuan Medical College Nanchong Sichuan China

**Keywords:** disorders of sex development, karyotype, SRY

## Abstract

We report an atypical disorders of sex development (DSD) case with no mutation of SYR gene but partial Yq deletion and partial duplication of Yp and Yq. This case emphasizes duplicated region Yp11.2→Yq11.223 with partial deletion of Yq11.223→Yqter most probably perturbed the sex differentiation and led to female phenotype.

## 1 INTRODUCTION

Disorders of sex development (DSD) are defined by congenital conditions in which the development of chromosomal, gonadal, or anatomical sex is atypical.^1^ DSD is a rare form of sex reversal in primary amenorrhea female with 46, XY karyotype.^2^ The chromosomal aberrations in lymphocytes of peripheral blood include Y chromosomes translocation with autosome^3^ and X chromosomes,^4^ and partial deletions/microdeletions of chromosome 9.^5^, ^6^ Typically genetic etiology of DSD is mutations of the sex‐determining region on the Y chromosome (SRY) gene. These mutations lead that SRY gene fails to upregulate the transcription of another key DSD gene and transcription factor SOX9 during early embryonic development. Then, initially immature bipotential gonads fail to differentiate along the male (testicular) pathway.^7^, ^8^ So far, clinical data have shown that 10%‐15% of DSD have SRY gene mutations including nearly 40 different mutations described in XY female patients.^9^

It is rare that the rearrangements of Y chromosome resulting in monocentric structure with duplication of large segments of short and long arms of Y chromosome and a partial deletion of Yq.^10^ Besides, these rare aberrance is present mostly in phenotype male.^11^ However, the most common cytogenetic aberrations of Y chromosome are isodicentric chromosomes in male and female cases.^12^, ^13^, ^14^ Phenotypes depend on the location of the breakpoints and on the proportion of aberrant cell lines which vary among male, abnormal female, and individual with ambiguous genitalia.^13^ Due to mitotic instability of idics, patients with idics always develop a mosaic with 45,X cell line, and with a wide spectrum of manifestations regardless of females with Turner symptoms and males with spermatogenic failure.^15^, ^16^ Furthermore, the breakpoints in these patients were found to locate in the 11.2 region of Yq which contains a azoospermic factor (AZF) region. It has been confirmed that most of the patients have AZF microdeletion.^14^

Here, we described a primary amenorrhea female who with a derivative Y chromosome [der(Y)] identified by standard cytogenetic analysis. To delineate this der(Y), fluorescence in situ hybridization (FISH) and SNP array characterization was performed. Another, the molecular analysis of the AZF was performed to characterize the aCGH results of Yq11.2 deletion.

## 2 METHODS

### 2.1 Clinical data

A 17‐year‐old girl was referred to the cytogenetic laboratory for chromosome analysis for primary amenorrhea. She exhibited a female appearance and voice, with little subcutaneous fat, no beard, or laryngeal prominence. And she exhibited female external genitalia, with hypogenetic labia majora and minora, sparse pubic hair, and a visible vaginal orifice. She was born following a full‐term normal delivery to nonconsanguineous parents, and her mother denied the use of any sex hormone drugs or exposure to radioactive substances during pregnancy. Her younger brother exhibited a normal phenotype.

The B‐mode ultrasound showed a promordial uterus, the left Ovarian cannot display, and the right adnexa display 28 × 24 mm cystic separation. Serum sex hormone analysis revealed that the follicle‐stimulating hormone level was 58.18 IU/L, the luteinizing hormone level was 18.17 IU/L, the estradiol level was 15 pg/mL, the prolactin level was 18.34 ng/mL, the pregnendione level was 0.3 ng/mL, and the testosterone level was 0.21 ng/mL.

All procedures used in the study confirmed to the tenets of the Declaration of Helsinki. The Ethics Committee of Affiliated Hospital of North Sichuan Medical College approved the protocols used. All participants have known to participate in the study. Written informed consents were obtained from all participants.

### 2.2 Karyotype analysis of G‐banding in lymphocytes

Chromosomal analysis of peripheral blood lymphocytes was performed according to standard protocols. Peripheral blood (2 mL) was collected in heparin vacutainers (Becton Dickinson, Franklin Lakes, NJ, USA). Cytogenetic studies were carried out on the patient, her parents, and her younger brother. For cytogenetic analysis, whole blood (0.5 mL) cultures were set up in 5 mL Roswell Park Memorial Institute (RPMI) 1640 media (GIBCO BRL, Grand Island, NY, USA) containing 15% fetal calf serum (GIBCO BRL, Grand Island, NY, USA), antibiotic mixture, and phytohemagglutinin P (GIBCO BRL, Grand Island, NY, USA) for 72 hours. Chromosome preparations were obtained from lymphocyte cultures and analyzed after Giemsa‐Trypsin‐Giemsa (GTG)‐banding. For karyotyping, at least 30 metaphases were analyzed, and for karyotypes of suspected mosaicism, 100 cells were counted. The karyotypes were interpreted using the recommendation of the International System for Human Cytogenetic Nomenclature.

### 2.3 Fluorescence in situ hybridization (FISH) in metaphase cells

Metaphase cells were performed on cultured, phytohemagglutinin (PHA)‐stimulated peripheral blood lymphocytes, according to standard cytogenetic procedures. FISH followed manufacturer’s instructions, using centromeric probes for chromosomes CSP 18/X/Y (18p11.1‐q11.1, Xp11.1‐q11.1, Yp11.1‐q11.1; GPMedical, Beijing, China). The slides were analyzed by two independent observers using an Olympus BX50 (Olympus Optical Co., Ltd., Tokyo, Japan) fluorescence microscope equipped with a filter set for FITC, Texas Red, and Aqua. Normal male lymphocyte preparations were used as controls for the FISH assays.

### 2.4 Multiplex quantitative fluorescent polymerase chain reaction (MQF‐PCR)

Genomic DNA from 200 μL peripheral blood of the propositus, her family members, a healthy fertile man, and a normal woman was extracted using blood DNA extraction Kits (TIANGEN, Beijing, China), using the procedure recommended by the producer. Amplification on 2 STR loci on chromosomes X (DXS981, DXS6809), one common STR loci on chromosomes X and Y, one gender specific loci AMXY, and three STR loci on chromosome 21 specific (D21S1435 D21S1411, D21S11) was conducted using 21‐trisome and sex chromosomes polyploid detection kit (Daan Gene, Guangzhou, China), PCR mix created as directed by the producer. The kit contains primer pairs for 4 markers on sex chromosomes, 3 markers for chromosome 21, all in a single multiplex reaction. Fragmental Analysis was conducted on an ABI 3130 Genetic Analyzer, using Run 3130 Data Collection software, using 36 cm capillary array length, and performance optimizing polymer (POP) 7. Run time was set to 1800 seconds. Sizing standard used was ABI LIZ 500. Data analysis and electropherogram creation were carried out using GeneMapper ID v3.2 software.

### 2.5 SNP array

Microdeletion/microduplication screening was performed using an SNP array platform (CytoScan HD SNP array; Affymetrix, Santa Clara, CA), following the manufacturer’s instructions. The CytoScan HD SNP array has 2.69 million probes, including 1.95 million copy number probes and 0.74 million SNP probes. Array data were analyzed using the Chromosome Analysis Suite (ChAS) software v 2.0 (Affymetrix).

### 2.6 Molecular analysis

Genomic DNA from peripheral blood of the propositus, her family members, one normal male volunteers, and one normal female volunteers was extracted using blood DNA extraction Kits (TIANGEN, Beijing, China). Sequences of the entire coding regions of SRY was amplified with primers shown in Table S1 and sequenced (Sangon Biotech Co., Ltd, Shanghai, China). The three discrete regions, AZFa, AZFb, and AZFc, located on the long arm of the Y chromosome, were performed by multiplex PCR (Polymerase Chain Reaction) amplification. The set of PCR primers (Table S1) (Sangon Biotech Co., Ltd, Shanghai, China) for the diagnosis of microdeletion of the AZFa, AZFb, and AZFc region included: sY84, sY86, sY127, sY134, sY254, sY255, SRY, and ZFX/ZFY.

## 3 RESULTS

**Figure 1:**
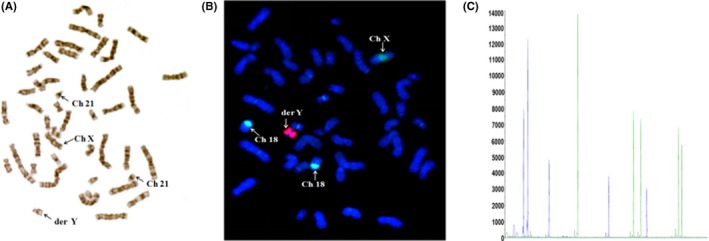
Results of karyotype, FISH and QF‐PCR. A, In G‐banded karyotype, the der (Y) in the patient were seen. B, Fluorescence in situ hybridization on metaphase chromosomes using Y (Yp11.1‐q11.1),X(Xp11.1‐q11.1), and 18 (18p11.1‐q11.1) chromosome centromere‐specific probes. Arrows indicate two Y chromosome specific centromeric signals (red) on one chromosome. C, Capillary electrophoresis results for the propositus’ sample with sex chromosomal aneuploidies (47, XYY) identified in the prospective examination. The results are produced using the GeneMapper software, showing full ranges and all sizes of the detected peaks on the vertical and horizontal axes, respectively, for intuitive intra‐sample visual comparison of the peaks. Fragment sizes are shown in bp on the horizontal axis. Arbitrary fluorescence units are shown on the vertical axis

Conventional cytogenetic analysis (G‐bands) revealed a der(Y) present in all 30 observed metaphase cells from the patient (Figure 1A). Because her parents and younger brother had normal G‐banded karyotypes, it was concluded that the der(Y) was a de novo aberration.

To confirm the der(Y), FISH analysis was performed with Y chromosome centromere probe in metaphase cells from the patient and her father and younger brother. Two adjacent red fluorescence singers (Y chromosome centromere probe) in one chromosome were found in the propositus’ sample (Figure 1B), but only one red fluorescence singer appeared in her father’ and younger brother’ sample, respectively. MQF‐PCR was also adopted to confirm the duplication of Y chromosome and the results showed the ratio of AMX (106) peak area to AMY(112) peak area was 0.5, and confirmed that the patient’s Y chromosome is duplicated (Figure 1C). Based on conventional analysis, the karyotype 46,X,dup(Y)(q11.22;p11.3) was expected.

**Figure 2:**
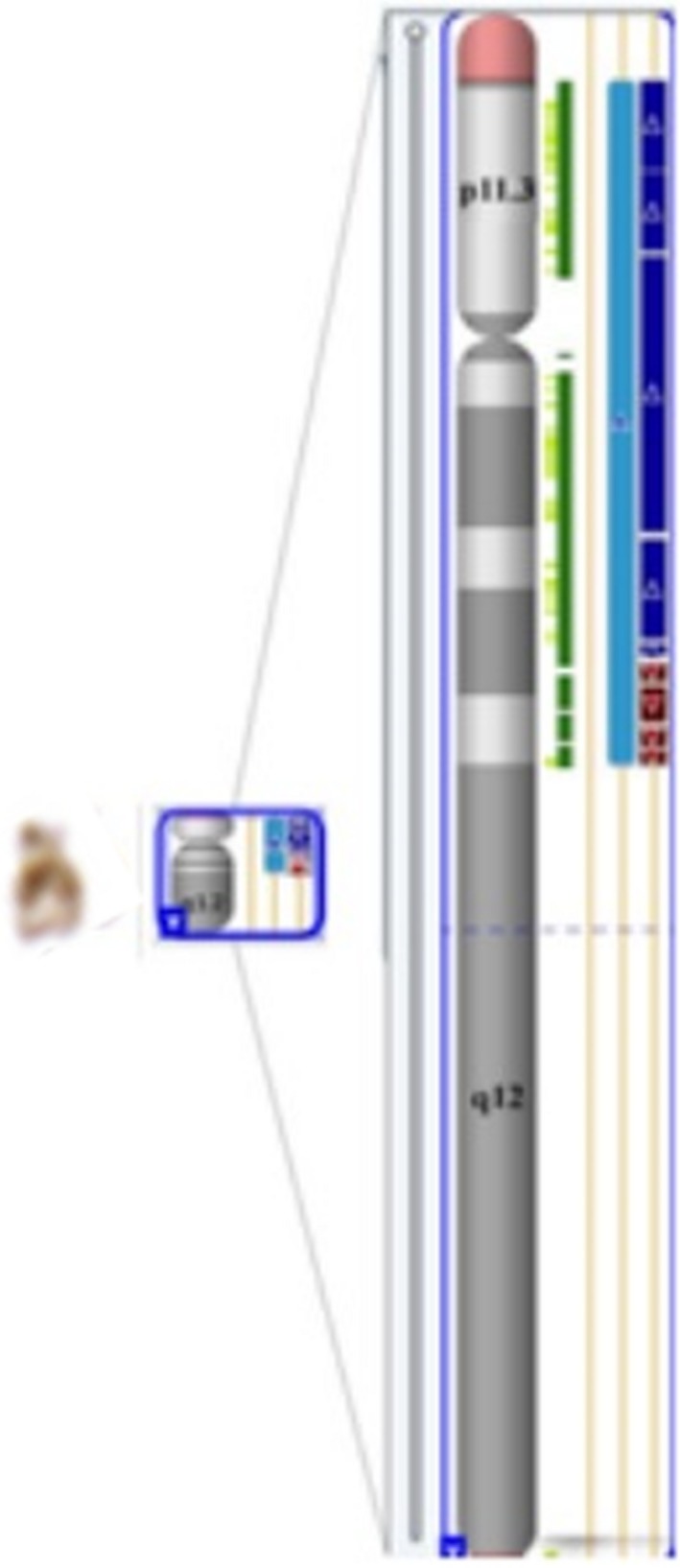
Deletion and duplication of der(Y) detected by CMA (CytoScan HD array). The array data showed that Yq11.23 region was deleted, while Yq11.223‐Yq11.23 and most of Yp arm were duplicated. The duplication region include Yp11.2q11.223 (2,650,424‐24,636,055)×2, and the deletion region include Yq11.223q11.23 (24,985,375‐28,799,654) ×0

Furthermore, in order to exclude a cryptic rearrangement of the Y chromosome and to test for potential genomic micro‐aberrations a genome‐wide study with CytoScan HD array was performed. The array data showed that Yq11.23 region was deleted, while Yq11.223‐Yq11.23 and most of Yp arm were duplicated (Figure 2 and Data S1). Thus, the patient’s karyotype has been identified as 46,X,der(Y) arr Yp11.2q11.223 (2,650,424‐24,636,055)×2, Yq11.223q11.23 (24,985,375‐28,799,654) ×0 according to the results of the aCGH analyses. We could not perform SNP array of her father and younger brother because they declined additional studies.

**Figure 3:**
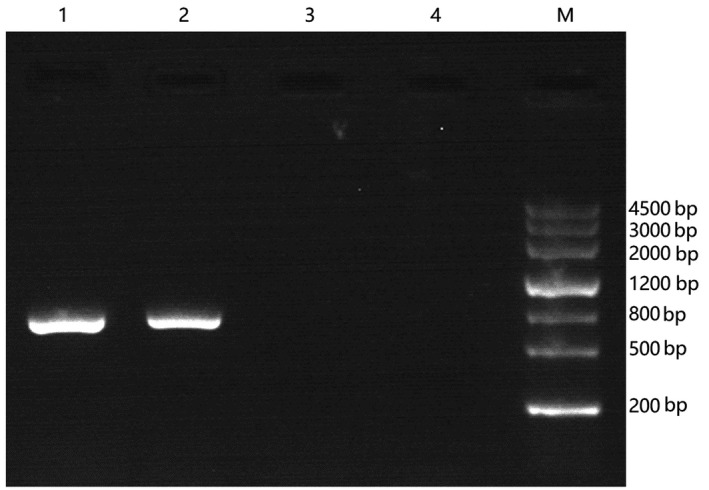
Result of SRY gene detected by polymerase chain reaction (PCR). Line 1: a DNA sample from the propositus; line 2: a DNA sample from a normal fertile man as a positive control; line 3: a DNA sample from a woman as a negative control; line 4: sterile water replaces DNA as no template control; M: Dmarker III

**Figure 4:**
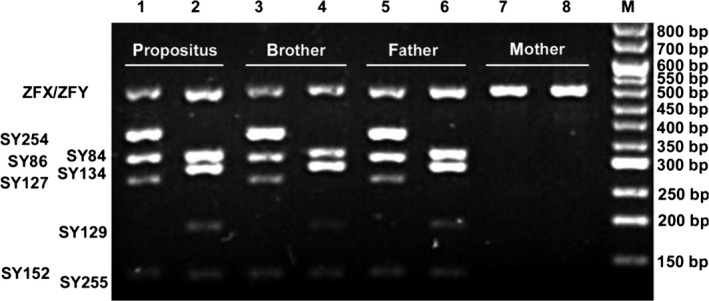
Result of SRY gene detected by polymerase chain reaction (PCR). Lines 1 and 2: DNA sample from the propositus; lines 3 and 4: DNA sample from the propositus’ younger brother; lines 5 and 6: DNA sample from the propositus’ father; lines 7 and 8: DNA sample from the propositus’ mother; M: DNA Marker I. the bands of the odd lines refer to ZFX/ZFY, SY254, SY86, SY127, and SY152 according to the fragments’ bps from long to short, respectively; the even‐numbered sets were ZFX/ZFY, SY84, SY134, SY129, and SY255

Molecular analysis revealed that the SRY gene was present with no mutation compared with her father and younger brother (Figure 3), and the AZFa, AZFb, and AZFc region were also present (Figure 4).

## 4 DISCUSSION

Disorders of sex development is a common aberration of the Y chromosome, and most cases are found in a mosaic form.^17^, ^18^, ^19^, ^20^ In our patient with primary amenorrhea and de novo derivative Y chromosome, this study’s approach demonstrated that this der(Y) is pseudoisotricentric partial of Yp‐Yq, where partial deletion of Yq occurred in concomitance with its replacement with inverted duplicated fragment of Y chromosome (p11.1‐q11.223) (see Figure 2D). The female phenotype in our patient might be a result of the relative absence of the 46,XY cell line in the reproductive system, with an inactivation of SRY due to duplication of Y chromosome (p11.1‐q11.223).

The deletion of the Yq11.223‐q11.23 contain multi‐gene which are important to spermatogenesis, that is, AZF(DAZ) and TTTY (Data S1). However, the molecular analysis results showed that the AZF was present. As the array data indicate that the duplicated region (Yp11.2q11.223) did not contain the AZF (DAZ), but most of the members of the family TTTY was present. The other region is mosaic normal copies region Yp11.31q11.23(2,650,424‐28,799,654) (Data S1), the AZF(DAZ) was present. There was no Y chromosome material in other chromosomes nor additional Y chromosome showed in the results of karyotype and FISH, and we presumed that the duplication of Y chromosome region is compressed on the same chromosome and then form a de novo derivative pseudoiso Y chromosome.

The primordial gonad is bipotential and can differentiate into a testis or an ovary, depending on the SRY gene located in the short arm of the Y chromosome. The absence of SRY permits the bipotential gonad to differentiate into an ovary at the eighth week of the embryo, leading to the female phenotype. The mutation, deletion, or translocation of SRY can affect the binding of the SRY proteins with DNA, and consequently contribute to sex reversal.^21^ The results of the molecular analysis in our patient showed that the SRY gene was present with no mutation (Figure S1). The results of array showed that SRY gene was also present in the duplication region and normal copies region, and the deletion region did not contain the SRY gene. Duplication or haploinsufficiency, such as DAX1, WNT4 or SOX9, SF1, WT1, and DMRT1‐DMRT2, has been considered responsible for the development of 46,XY sex reversal,^22^, ^23^ and therefore, the duplication of SRY gene in our patient may play a significant role in the etiology of the disease in this case.

A de novo karyotype 46,X,del(Y)(q11.2) observed in primary amenorrhea female after FISH and aCGH analysis permitted better characterization of derivative Y chromosome with final karyotype description as 46,X,der(Y)del(Y)(q11.2). dup (p11.1→q11.1).ish. arr Yp11.2q11.223(2,650,424‐24,636,055)×2, Yq11.223q11.23 (24,985,375‐28,799,654)×0, Yp11.31q11.23(2,650,424‐28,799,654)×1‐2. Finally, duplicated region Yp11.2→Yq11.223 with partial deletion of Yq11.223→Yqter most probably perturbed the sex differentiation and led to female phenotype.

## CONFLICT OF INTEREST

The authors declare that they have no competing interests.

## AUTHORSHIP

QL and XZ: wrote the manuscript and collected the clinical data and were involved in scientific input and revision. QM and CH: involved in scientific input and revision. JS: is the senior author and was involved in scientific input and revision of the final manuscript.
