# Supplementary figures and images for: A de novo derivative Y chromosome (partial Yq deletion and partial duplication of Yp and Yq) in a female with disorders of sex development
Source: Clin Case Rep. 2018 Jul 7;6(9):1671–6. doi: 10.1002/ccr3.1613 (PMC6132170; doi:10.1002/ccr3.1613)

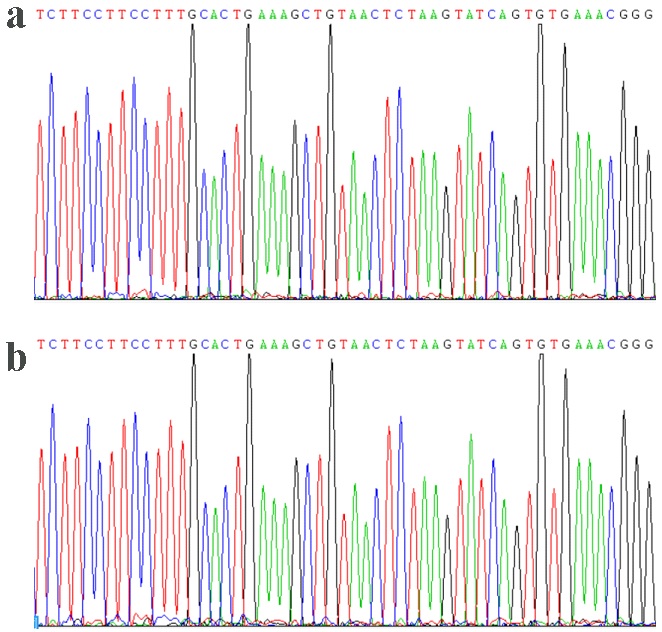

Supplement: Supplementary file 1 [file CCR3-6-1671-s001.jpg]
