# Supplementary material for: A de novo derivative Y chromosome (partial Yq deletion and partial duplication of Yp and Yq) in a female with disorders of sex development
Source: Clin Case Rep. 2018 Jul 7;6(9):1671–6. doi: 10.1002/ccr3.1613 (PMC6132170; doi:10.1002/ccr3.1613)
Supplement: Supplementary file 2 [file CCR3-6-1671-s002.docx]

**Supplementary table 1. Primers sequences of the genes used in PCR**

| gene |  | Primers sequences(5'-3') | Size(bp) |
| --- | --- | --- | --- |
| SRY | formal | CGAACTCTGGCACCTTTCAA | 677 |
|  | reverse | GTTACCCGATTGTCCTACAGCT |  |
| SY254 | formal | GAACCGTATCTACCAAAGCAGC | 400 |
|  | reverse | GGGTGTTACCAGAAGGCAAA |  |
| SY86 | formal | GTGACACACAGACTATGCTTC | 320 |
|  | reverse | ACACACAGAGGGACAACCCT |  |
| SY127 | formal | GGCTCACAAACGAAAAGAAA | 274 |
|  | reverse | CTGCAGGCAGTAATAAGGGA |  |
| SY152 | formal | ACAGGAGGGTACTTAGCAGT | 125 |
|  | reverse | AAGACAGTCTGCCATGTTTCA |  |
| SY84 | formal | AGAAGGGTCTGAAAGCAGGT | 326 |
|  | reverse | GCCTACTACCTGGAGGCTTC |  |
| SY134 | formal | GTCTGCCTCACCATAAAACG | 301 |
|  | reverse | ACCACTGCCAAAACTTTCAA |  |
| SY255 | formal | GTTACAGGATTCGGCGTGAT | 126 |
|  | reverse | CTCGTCATGTGCAGCCAC |  |
| SY129 | formal | AGCTTCAGGAGGTTCAAAAC | 201 |
|  | reverse | AAGTGGGACCTAAGCTACGA |  |
| ZFX/ZFY | formal | ACCRCTGTACTGACTGTGATTACAC | 495 |
|  | reverse | GCACYTCTTTGGTATCYGAGAAAGT |  |
