# Supplementary material for: A de novo derivative Y chromosome (partial Yq deletion and partial duplication of Yp and Yq) in a female with disorders of sex development
Source: Clin Case Rep. 2018 Jul 7;6(9):1671–6. doi: 10.1002/ccr3.1613 (PMC6132170; doi:10.1002/ccr3.1613)
Supplement: Supplementary file 3 [file CCR3-6-1671-s003.pdf]

Segments Table

For research use only. Not for use in diagnostic procedures.

Selected Results From: ChrY

Interpretation

wuquxua-Y

Data Files In View

File Label	File Name
20141225_104102_007_20141225-7.cy750K.cychp	20141225_104102_007_20141225-7.cy750K.cychp

Genome Filters

Segment Type	Filter Type	Minimum Filter Value
Gain	Segment Length	200000
Loss	Segment Length	200000
GainMosaic	Segment Length	200000
LossMosaic	Segment Length	200000
LOH	Segment Length	1000000

Settings for 20141225_104102_007_20141225-7.cy750K.cychp

Setting Type	Setting
Smoothing	On

Setting Type	Setting
Smoothing Max Jump Limit	Off
Joining	25 Markers, 200 kbp
Joining Max Jump Limit	Off
Restricted Mode	Off
QC Metrics	mapd ≤ 0.25; snpQC ≥ 15; wavinessSd ≤ 0.12

Microarray Nomenclature

20141225_104102_007_20141225-7.cy750K.cychp:
arr[hg19] Yq11.223(24,253,907-24,636,055)x2, Yp11.2(6,132,242-9,175,516)x2, Yp11.31p11.2(2,650,424-6,112,588)x2, Yq11.222q11.223(20,235,418-24,020,509)x2, Yp11.2q11.222(9,369,244-19,942,667)x2, Yq11.23(28,320,667-28,799,654)x0, Yq11.223(24,985,375-25,629,481)x0, Yq11.23(27,448,830-28,105,479)x0, Yq11.223q11.23(25,863,808-27,224,389)x0, Yp11.31q11.23(2,650,424-28,799,654)x1-2

Segments Details

File	CN State	Type	Chromosome	Cytoband Start	Size (kbp)	Marker Count	Gene Count	Genes	OMIM® Genes Count	OMIM® Genes	CytoRegions	Call	Interpretation	Call & Interpretation By	Materially Modified Segment	Microarray Nomenclature
20141225_104102_007_20141225-7.cy750K.cychp	2.0	Gain	Y	q11.223	382.148	77	6	TTTY6, TTTY6B, RBMY1J, RBMY1F, TTTY5, RBMY2FP	2	TTTY6 (400039), TTTY5 (400038)	Cytoregions Not Set				false	arr[hg19] Yq11.223(24,253,907-24,636,055)x2

File	CN State	Type	Chromosome	Cytoband Start	Size (kbp)	Marker Count	Gene Count	Genes	OMIM® Genes Count	OMIM® Genes	CytoRegions	Call	Interpretation	Call & Interpretation By	Materially Modified Segment	Microarray Nomenclature
20141225_104102_007_20141225-7.cy750K.cychp	2.0	Gain	Y	p11.2	3043.274	656	20	TTTY1B, TTTY1, TTTY2, TTTY2B, TTTY21B, TTTY21, TTTY7, TTTY7B, TTTY8, TTTY8B, AMELY, TBL1Y, PRKY, TTTY16, TTTY12, TTTY18, TTTY19, TTTY11, RBMY1A3P, TTTY20	3	AMELY (410000), TBL1Y (400033), PRKY (400008)	Cytoregions Not Set				false	arr[hg19] Yp11.2(6,132,242-9,175,516)x2
20141225_104102_007_20141225-7.cy750K.cychp	2.0	Gain	Y	p11.31	3462.164	385	8	SRY, RPS4Y1, ZFY, LINC00278, TGIF2LY, PCDH11Y, TTTY23, TTTY23B	5	SRY (480000), RPS4Y1 (470000), ZFY (490000), TGIF2LY (400025), PCDH11Y (400022)	Cytoregions Not Set				false	arr[hg19] Yp11.31p11.2(2,650,424-6,112,588)x2

File	CN State	Type	Chromosome	Cytoband Start	Size (kbp)	Marker Count	Gene Count	Genes	OMIM ® Genes Count	OMIM ® Genes	CytoRegions	Call	Interpretation	Call & Interpretation By	Materially Modified Segment	Microarray Nomenclature
20141225_104102_007_20141225-7.cy750K.cychp	2.0	Gain	Y	q11.222	3785.091	658	25	XKRY2, XKRY, LINC00230A, LINC00230B, FAM41AY2, FAM41AY1, HSFY2, HSFY1, TTTY9B, TTTY9A, NCRNA00185, TTTY14, CD24, BCORP1, TXLNG2P, KDM5D, TTTY10, EIF1AY, RPS4Y2, RBMY2EP, RBMY1B, RBMY1A1, RBMY1D, RBMY1E, TTTY13	6	HSFY1 (400029), CD24 (600074), TXLNG2P (400031), KDM5D (426000), EIF1AY (400014), RPS4Y2 (400030)	Cytoregions Not Set				false	arr[hg19] Yq11.222q11.223(20,235,418-24,020,509)x2

File	CN State	Type	Chromosome	Cytoband Start	Size (kbp)	Marker Count	Gene Count	Genes	OMIM® Genes Count	OMIM® Genes	CytoRegions	Call	Interpretation	Call & Interpretation By	Materially Modified Segment	Microarray Nomenclature
20141225_104102_007_20141225-7.cy750K.cychp	2.0	Gain	Y	p11.2	10573.423	1196	30	RBMY3AP, TTTY8, TTTY8B, TTTY7, TTTY7B, TTTY21B, TTTY21, TTTY2B, TTTY2, TTTY1, TTTY1B, TTTY22, TTTY23, TTTY23B, GYG2P1, TTTY15, USP9Y, DDX3Y, UTY, TMSB4Y, VCY1B, VCY, NLGN4Y, NLGN4Y-AS1, FAM41AY2, FAM41AY1, LINC00230B, LINC00230A, XKRY2, XKRY	7	USP9Y (400005), DDX3Y (400010), UTY (400009), TMSB4Y (400017), VCY (400012), NLGN4Y (400028), XKRY (400015)	Cytoregions Not Set				false	arr[hg19] Yp11.2q11.222(9,369,244-19,942,667)x2

File	CN State	Type	Chromosome	Cytoband Start	Size (kbp)	Marker Count	Gene Count	Genes	OMIM ® Genes Count	OMIM ® Genes	CytoRegions	Call	Interpretation	Call & Interpretation By	Materially Modified Segment	Microarray Nomenclature
20141225_104102_007_20141225-7.cy750K.cychp	0.0	Loss	Y	q11.23	478.987	31	0		0		Cytoregions Not Set				false	arr[hg19] Yq11.23(28,320,667-28,799,654)x0
20141225_104102_007_20141225-7.cy750K.cychp	0.0	Loss	Y	q11.223	644.106	40	13	TTTY17B, TTTY17C, TTTY17A, TTTY4, TTTY4C, TTTY4B, BPY2C, BPY2, BPY2B, DAZ1, DAZ4, DAZ3, DAZ2	3	TTTY17A (400040), TTTY4 (400037), DAZ1 (400003)	Cytoregions Not Set				false	arr[hg19] Yq11.223(24,985,375-25,629,481)x0
20141225_104102_007_20141225-7.cy750K.cychp	0.0	Loss	Y	q11.23	656.649	100	7	GOLGA2P3Y, GOLGA2P2Y, CSPG4P1Y, CDY1, CDY1B, TTTY3B, TTTY3	1	TTTY3 (400036)	Cytoregions Not Set				false	arr[hg19] Yq11.23(27,448,830-28,105,479)x0

File	CN State	Type	Chromosome	Cytoband Start	Size (kbp)	Marker Count	Gene Count	Genes	OMIM® Genes Count	OMIM® Genes	CytoRegions	Call	Interpretation	Call & Interpretation By	Materially Modified Segment	Microarray Nomenclature
20141225_104102_007_20141225-7.cy750K.cychp	0.0	Loss	Y	q11.223	1360.581	144	19	TTTY3B, TTTY3, CDY1, CDY1B, CSPG4P1Y, GOLGA2P3Y, GOLGA2P2Y, TTTY17B, TTTY17C, TTTY17A, TTTY4C, TTTY4, TTTY4B, BPY2, BPY2C, BPY2B, DAZ4, DAZ3, DAZ2	6	CDY1 (400016), CSPG4P1Y (400034), GOLGA2P2Y (400035), BPY2 (400013), DAZ3 (400027), DAZ2 (400026)	Cytoregions Not Set				false	arr[hg19] Yq11.223q11.23(25,863,808-27,224,389)x0

File	CN State	Type	Chromosome	Cytoband Start	Size (kbp)	Marker Count	Gene Count	Genes	OMIM® Genes Count	OMIM® Genes	CytoRegions	Call	Interpretation	Call & Interpretation By	Materially Modified Segment	Microarray Nomenclature
20141225_104102_007_20141225-7.cy750K.cychp	1.1165582	GainMosaic	Y	p11.31	26149.23	4135	102	SRY, RPS4Y1, ZFY, LINC00278, TGIF2LY, PCDH11Y, TTTY23, TTTY23B, TSPY2, TTTY1B, TTTY1, TTTY2, TTTY2B, TTTY21B, TTTY21, TTTY7, TTTY7B, TTTY8, TTTY8B, AMELY, TBL1Y, PRKY, TTTY16, TTTY12, TTTY18, TTTY19, TTTY11, RBMY1A3P, TTTY20, FAM197Y2P, FAM197Y5, TSPY8, TSPY4, TSPY3, TSPY1, RBMY3A	38	SRY (480000), RPS4Y1 (470000), ZFY (490000), TGIF2LY (400025), PCDH11Y (400022), AMELY (410000), TBL1Y (400033), PRKY (400008), TSPY1 (480100), USP9Y (400005), DDX3Y (400010), UTY (400009), TMSB4Y (400017), VCY (400012), NLGN4Y (400028), XKRY (400015), CDY2A (400018), HSFY1 (400029), CD24 (600074)	Cytoregions Not Set				false	arr[hg19] Yp11.31q11.23(2,650,424-28,799,654)x1-2

File	CN State	Type	Chromosome	Cytoband Start	Size (kbp)	Marker Count	Gene Count	Genes	OMIM ® Genes Count	OMIM ® Genes	CytoRegions	Call	Interpretation	Call & Interpretation By	Materially Modified Segment	Microarray Nomenclature
								RBMY3AP, TTTY22, GYG2P1, TTTY15, USP9Y, DDX3Y, UTY, TMSB4Y, VCY1B, VCY, NLGN4Y, NLGN4Y-AS1, FAM41AY2, FAM41AY1, LINC00230B, LINC00230A, XKRY2, XKRY, CDY2B, CDY2A, HSFY2, HSFY1, TTTY9B, TTTY9A, NCRNA00185, TTTY14, CD24, BCORP1, TXLNG2P, KDM5D, TTTY10, EIF1AY, RPS4Y2,		(600074), TXLNG2P (400031), KDM5D (426000), EIF1AY (400014), RPS4Y2 (400030), RBMY1A1 (400006), PRY (400019), PRY2 (400041), TTTY6 (400039), TTTY5 (400038), TTTY17A (400040), TTTY4 (400037), DAZ1 (400003), CDY1 (400016), CSPG4P1Y (400034), GOLGA2P2Y (400035), BPY2 (400013)						

File	CN State	Type	Chromosome	Cytoband Start	Size (kbp)	Marker Count	Gene Count	Genes	OMIM ® Genes Count	OMIM ® Genes	CytoRegions	Call	Interpretation	Call & Interpretation By	Materially Modified Segment	Microarray Nomenclature
								RPS4Y2, RBMY2EP, RBMY1B, RBMY1A1, RBMY1D, RBMY1E, TTTY13, PRY, PRY2, TTTY6, TTTY6B, RBMY1J, RBMY1F, TTTY5, RBMY2FP, TTTY17B, TTTY17C, TTTY17A, TTTY4, TTTY4C, TTTY4B, BPY2C, BPY2, BPY2B, DAZ1, DAZ4, DAZ3, DAZ2, TTTY3B, TTTY3, CDY1, CDY1B, CSPG4P1Y,		(400013), DAZ3 (400027), DAZ2 (400026), TTTY3 (400036)						

File	CN State	Type	Chromosome	Cytoband Start	Size (kbp)	Marker Count	Gene Count	Genes	OMIM ® Genes Count	OMIM ® Genes	CytoRegions	Call	Interpretation	Call & Interpretation By	Materially Modified Segment	Microarray Nomenclature
								1Y, GOLGA2P3Y, GOLGA2P2Y								
